# Single-cell transcriptomics of granulocytes in asthma and atopic diseases

**DOI:** 10.3389/fmolb.2026.1739145

**Published:** 2026-06-02

**Authors:** Nicholas T. Hogan, Alexis Garduno, Francisco Santos, Eugenia Winata, John-Wesley Pabalate, Praveen Akuthota, Gregory Seumois

**Affiliations:** 1 San Diego Biomedical Research Institute (SDBRI), San Diego, CA, United States; 2 University of California San Diego, San Diego, CA, United States

**Keywords:** asthma, atopic disease, basophils, eosinophils, granulocytes, mast cells, neutrophils, scRNAseq

## Abstract

Since their early association with atopic disease, granulocytes, particularly eosinophils, have been widely used as biomarkers, yet the mechanisms underlying their role in airway and atopic inflammatory diseases remain incompletely understood. Single-cell transcriptomic approaches have begun to reveal substantial phenotypical and functional heterogeneity among granulocytes such as neutrophils and eosinophils, as well as in mast cells. In contrast, basophils remain sparsely represented in current datasets. As studies of other immune lineages have shown the significance of cellular diversity in atopic disease, extending this resolution to granulocytes marks an important frontier. In this review, we outline the historical and technical challenges that have constrained single-cell studies of human granulocytes and highlight the methodological breakthroughs that are now overcoming these barriers. We systematically reviewed published granulocyte scRNA-seq studies using PubMed and Talk2Data (BioTuring) platform. Our review underscores emerging roles for granulocyte heterogeneity in asthma and atopic disease and identifies remaining knowledge gaps where experimental and computational advances may accelerate understanding of their pathophysiologic functions.

## 1 Introduction

Despite their early recognition by 19th-century pioneers of microscopy (Kay, 2015), granulocytes were previously viewed as monolithic first responders to infection, only recognized relatively recently for their potential for functional heterogeneity within the immune system (Lin and Loré, 2017). This contrasts with lymphocytes and other myeloid innate immune cells, which were recognized much earlier as corresponding to a multitude of phenotypic and functional subpopulations. Indeed, these subpopulations of non-granulocyte immune cells have long been recognized as key mechanistic players in diverse pathologies, including infection, cancer, autoimmunity, and atopic diseases.

Despite this, granulocytes have long been associated with atopic diseases, especially asthma (Nelson et al., 2020). For instance, eosinophils have been associated with the asthmatic airway for over a century with sputum eosinophil count recognized as a biomarker which can guide response to systemic steroid therapy since the mid-20th century (Morrow Brown, 1958). Granulocytes are also important mediators of diseases and key targets for therapy, as the success of IL5-depleting therapies has demonstrated in conditions such as eosinophilic asthma and eosinophilic granulomatosis with polyangiitis (EGPA) (Ortega et al., 2014; Wechsler et al., 2017; Wechsler et al., 2024; Fong et al., 2021; Jayne et al., 2024). Yet while such therapies are beneficial to many patients, they are not universally effective (Bello et al., 2022; Kelly et al., 2017), raising the possibility that granulocyte populations themselves may differ amongst patients and disease endotypes. Granulocytes broadly, and especially eosinophils and neutrophils, remain key areas of focus in the field of atopic disease, and single-cell studies of these key players offer promise of improved disease endotyping, diagnosis, and therapy.

Single cell transcriptomics has facilitated the delineation of important subsets of immune cells such as T cells in multiple pathologies including atopic disease (Seumois and Vijayanand, 2019; Seumois et al., 2020; Herrera-De La Mata et al., 2023), and thus this method holds great promise in similarly identifying granulocyte heterogeneity of pathophysiologic importance. As we will discuss, the field is beginning to fulfill that promise. While bulk RNA-sequencing has enabled successful study of granulocytes (Hogan et al., 2024; Nelson et al., 2019), until quite recently, analogous studies using single-cell transcriptomics have been hampered by several challenges such as fragility and short-lived nature of granulocytes, their relatively low levels of mRNA, and the propensity for degradation of this RNA through presence of high levels of endogenous enzymes (Chhiba and Kuang, 2024).

Fortunately, recent years have seen the debut of granulocytes in single-cell transcriptomic atlases. However, scRNAseq databases for human granulocytes still lag behind other immune cell types, including in the field of atopic disease. Here we review the challenges of working with human granulocytes, the techniques which have brought early successes over these challenges and resulting insights from these initial transcriptomic analyses into the biology of human neutrophils, eosinophils, basophils, and mast cells in atopic disease.

### 1.1 A brief overview of single cell transcriptomic methods

As scRNA-seq technology has advanced, two major techniques have come to predominate in large-scale studies detailing immune cell subsets, droplet-based and microwell-based scRNAseq. The droplet-based technique involves emulsifying single cells in oil where they are barcoded (Macosko et al., 2015; Klein et al., 2015), allowing for single-cell resolution of downstream RNA-sequencing. This technology has been made widely available by 10X Genomics (Luecken and Theis, 2019). Capture of mRNA can be performed from the 5′ or 3′ ends, and the Flex protocol supports fixation of cells prior to processing (Llora-Batlle et al., 2024), through a probe-based capture approach, improving the ability to multiplex samples. This approach is highly efficient in recovering lymphoid and mononuclear phagocyte populations, generating high transcript counts per cell and enabling a range of downstream analyses (Luecken and Theis, 2019), including RNA velocity (La Manno et al., 2018) and trajectory inference (Rodríguez-González et al., 2024; Trapnell et al., 2014). More recently, droplet-based platforms have been adapted for multi-omics approaches (Hao et al., 2021; Ma et al., 2020; Stoeckius et al., 2017), allowing paired measurements of transcriptomes, chromatin accessibility, and surface proteomes, which can better resolve granulocyte activation states and their signaling networks.

Microwell-based approaches (Gierahn et al., 2017) (e.g., BD Rhapsody (BD Rhapsody TM, 2026)) represent a prominent alternative methodology. This approach allows gravity-driven cells to settle into wells with barcoded capture beads, and it has been implemented successfully to recover eosinophils and other fragile cell types in murine models (Borrelli et al., 2024). Borrelli and colleagues describe this approach and hypothesize that microwell-based methods are gentler, causing less shear stress than alternative scRNA-seq methods, enabling improved recovery of transcriptomes of fragile cells such as granulocytes (Borrelli et al., 2024). Gravity-based scRNA-seq was recently applied to profile blood and tissue-resident eosinophils in eosinophilic esophagitis, revealing distinct transcriptional states that were not detected in droplet-based datasets (Ben-Baruch Morgenstern et al., 2024). Direct comparisons of droplet-based versus microwell-based scRNA-seq are limited, but one such study utilizing bronchoalveolar lavage samples demonstrated that while droplet-based methods provided higher gene counts per cell, microwell-based methods uniquely captured eosinophils, that were otherwise lost, highlighting platform-specific strengths (Janssen et al., 2025).

As the field advances, other techniques may become of interest in the study of granulocyte transcriptomic and functional heterogeneity, including particle-templated instant partition sequencing (PIP-seq) (Clark et al., 2023), for which commercial analytical platforms are now available (Fluentbio, 2026). There are now also methods which enable whole genome amplification (scWGA) (Zong et al., 2012) and whole gene body long RNA-sequencing (Hagemann-Jensen et al., 2020) at the single cell level, which are also emerging, although their application to granulocytes remains limited.

### 1.2 Granulocyte instability and processing artifacts

Granulocytes, neutrophils, eosinophils, and basophils, are integral to innate immunity and inflammation. However, despite their essential role, granulocytes remain among the most technically challenging immune cell populations to study due to a range of biological and technical constraints. We summarize additional challenges here.

#### 1.2.1 Short lifespan and cryopreservation limitations

**FIGURE 1:**
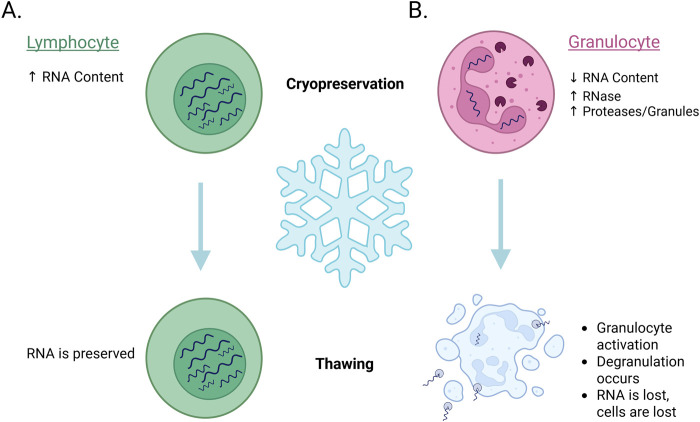
Lymphocytes and Granulocytes Have Differential Tolerance to Cryopreservation. Lymphocytes possess relatively high mRNA content, and transcripts are relatively preserved through cryopreservation and subsequent processing **(A)**. Conversely, granulocytes possess lower mRNA content, and cryopreservation triggers activation and degranulation, preventing capture of transcripts **(B)**.

The structural integrity of granulocytes deteriorates significantly following cryopreservation, with loss of membrane integrity (McCarthy et al., 1981; McCarthy et al., 1984; Takahashi et al., 1985), rendering them unsuitable for functional assays or single-cell RNA sequencing studies (Figure 1). Experimental data comparing different cryopreservation methods showed that granulocytes stored using traditional DMSO-based freezing media retained about 50% functionality, including processes such as phagocytosis and myeloperoxidase activity, after 2 months storage, but then showed progressive loss of viability and functionality thereafter (Boonlayangoor et al., 1980). Comparative analysis of cryopreserved bronchoalveolar lavage cells processed via droplet- and microwell-based scRNA-seq showed that neutrophils were recoverable across both methods, whereas eosinophils were preserved only in microwell workflows, underscoring the heightened fragility of this subset (Janssen et al., 2025). These findings underscore the necessity for real-time sample processing, as delayed handling can be significantly detrimental to the quality of resulting data. Current best practice recommendations from 10X Genomics suggest processing and loading cells or nuclei of interest onto the 10X Genomics chip within about 1 h (10xgenomics, 2026a) and immediate processing for granulocytes (10xgenomics, 2026b), while Goss et al. (2025) in their study of human blood eosinophils in asthma emphasize a risk of failed sequencing runs when eosinophil isolation takes longer than 2 h.

To address this challenge, fixation-based stabilization methods have been explored, in which chemical fixation (such as methanol (Alles et al., 2017) or paraformaldehyde) is used to preserve cellular morphology and RNA integrity. Protocols employing this method include 10X Flex, and examples of its effective implementation have been reported, including in a comparative study of single-cell transcriptomic methods using human blood neutrophils (Hatje et al., 2025). Because fixation effectively prevents post-collection cells and RNA degradation, this approach holds promise to study of granulocyte single-cell transcriptomics.

#### 1.2.2 Granulocyte RNA content and endogenous RNase activity

Granulocytes relatively contain lower levels of RNA compared to other immune cell lineages (Monaco et al., 2019), and this presents a significant barrier to transcriptomic studies. Nevertheless, as shown in a study of COPD, neutrophils were found to exhibit distinct molecular states despite their low RNA abundance, detectable only through optimized single-cell workflows coupled with high-sensitivity computational imputation (Kapellos et al., 2023). Targeted transcript enrichment strategies have also been explored, including Programmable Enrichment via RNA FlowFISH by sequencing (PERFF-seq), which selectively amplifies cellular subset-specific genes (Abay et al., 2025) and has been validated in peripheral blood mononuclear cells (PBMC). This technique could prove useful in the characterization of rare granulocyte sub-populations in future studies. However, while such techniques improve transcript recovery, they rely on predefined gene panels and therefore limit unbiased discovery.

RNA degradation is a general concern in transcriptomics studies (Jobarteh et al., 2014). This risk is particularly relevant for granulocytes, especially eosinophils (Yang and Miller, 1985; Rosenberg and Rosenberg, 2015), wich contain pre-formed RNA-degrading enzymes (Brinkmann et al., 2004; Rosenberg, 2008) within their cytoplasmic granules (i.e., RNase 2 [Eosinophil-Derived Neurotoxin, EDN], RNase3 [eosinophil cationic protein]), whose release during granule disruption will contribute to rapid RNA degradation during sample processing. Moreover, granulocytes have a short lifespan and once removed from their physiological environment, can rapidly initiate cell death pathways such as apoptosis or, upon activation, NETosis. These processes may further promote the release and activation of nucleases and RNases, thereby increasing RNA degradation during sample handling.

Relevant to our disease context, these granules are in fact released into the airway in asthma (Lu et al., 2018). Thus, when considering the study of granulocytes in atopic diseases, specifically airways diseases, extracellular RNase provide another hurdle in the pursuit of obtaining high scRNA-seq data.

To address this challenge, single-nucleus RNA sequencing (snRNA-seq) has been proposed as an alternative approach, although it provides reduced transcript coverage and an incomplete representation of the granulocyte transcriptome (Kim et al., 2023). Attempts to mitigate RNA degradation include the use of recombinant or synthetic RNase inhibitors during sample processing, which can improve RNA integrity and transcript recovery in scRNA-seq experiments (Hatje et al., 2025). However, these inhibitors do not fully neutralize RNase activity. Direct RNA stabilization using lysis buffers such as RNAprotect has also been explored (Riesgo et al., 2012). In practice, fixation-based stabilization methods remain one of the most reliable strategies to preserve granulocyte RNA for transcriptomic analysis.

#### 1.2.3 Granulocyte biology and handling challenges

Granulocytes are prone to mechanical and enzymatic stress during sample handling, leading to activation, degranulation, and NET formation. This presents a significant challenge for cell isolation and downstream procedures. For instance, a standard assay is cell immunophenotyping by fluorescence-activated cell sorting (FACS) technique. It has been shown that FACS induces stress responses that alter granulocyte behavior and alter granulocyte functions (Hundhammer et al., 2022; Andrä et al., 2020). This highlights the sensitivity of granulocytes not only to handling *ex vivo* but also to paracrine cues *in vivo* and raises the concern that *ex vivo* processing could trigger a response similar to true, disease-relevant biological signals. Gentler isolation methods, such as negative selection using magnetic bead-based depletion, have been proposed to improve granulocyte viability while minimizing activation-induced artifacts (Blanter et al., 2022). Additionally, DNase I treatment is often used to counteract NET-mediated cell clumping, enhancing the quality of single-cell suspensions, a mechanism which is also of therapeutic interest (Reichard and Asosingh, 2018; Wang et al., 2024). However, these approaches require extensive optimization, and no standardized protocol exists for the optimal isolation of granulocytes for downstream applications.

#### 1.2.4 Computational challenges and opportunities

The aforementioned challenges have slowed progress in granulocyte single cell transcriptomics as compared to other immune cells. However, bulk RNA-seq has proven successful in the study of *ex vivo* human eosinophils (Hogan et al., 2024), in a model of e-cigarette exposure, and neutrophils in comparative analyses of chronic autoimmune and inflammatory diseases and comparisons of murine and human cells (Hackert et al., 2023). As the field of single cell omics matures, computational approaches are also advancing, allowing researchers to leverage multi-omics to dissect granulocyte heterogeneity across multiple modalities despite technical barriers. For instance, benchmarking analyses have demonstrated that algorithms such as totalVI (total Variational Inference) and UINMF (Unshared Integrative Non-negative Matrix Factorization) showed promise in their ability to integrate transcriptomic data with protein and chromatin data (Gayoso et al., 2021; Kriebel and Welch, 2022). Additionally, methods have also been developed to meet challenges such as multiplet removal across multi-omic datasets, which is essential to accurately analyze single cell data (Hu et al., 2024). These tools are critical as scRNA-seq increasingly reveals granulocyte subsets with distinct transcriptional programs, such as interferon-responsive eosinophils in eosinophilic esophagitis or glycoRNA-bearing neutrophils in sterile inflammation (inflammation occurring in the absence of infection) (Ben-Baruch Morgenstern et al., 2024; Zhang et al., 2024). Integrative multi-omics analyses will help further study granulocyte transcriptional heterogeneity in health and disease.

## 2 Granulocyte single-cell transcriptomics in atopic disease: a nascent field

Granulocytes have recently made their debut in single-cell atlases pertaining to diseases including COPD, cancer, COVID-19, inflammatory bowel disease, as well as some murine disease models (Kapellos et al., 2023; Gurtner et al., 2023; Wu et al., 2024; Ramirez et al., 2024). However, their study in atopic diseases remains in its nascence, though it is an area of intense research interest. To date, neutrophils represent the granulocyte population most thoroughly investigated in single-cell transcriptomic studies overall. Wigerblad et al. published pioneering data on human peripheral blood neutrophils in 2022, revealing four distinct transcriptional states in blood, which they interpreted respectively to be immature, transitional, low transcriptional activity, and type I interferon-inducible neutrophils (Wigerblad et al., 2022). The largest single-cell study of human granulocytes comes from the cancer literature. In 2024, Wu and colleagues investigated tumor-associated neutrophil heterogeneity with their study examining human neutrophils in cancer, analyzing 225 samples from 143 patients with 17 different types of cancer using droplet-based scRNA-seq (Wu et al., 2024). They identified and functionally validated 10 distinct neutrophil subsets with transcriptional programs characterized by inflammation, angiogenesis, and antigen presentation, amongst others (Wu et al., 2024). These two studies represent important landmarks in the investigation of human granulocyte single cell transcriptomics and offer context for our discussion of granulocytes in atopic disease.

### 2.1 A systematic overview of scRNA-seq studies of granulocytes in atopic disease

**FIGURE 2:**
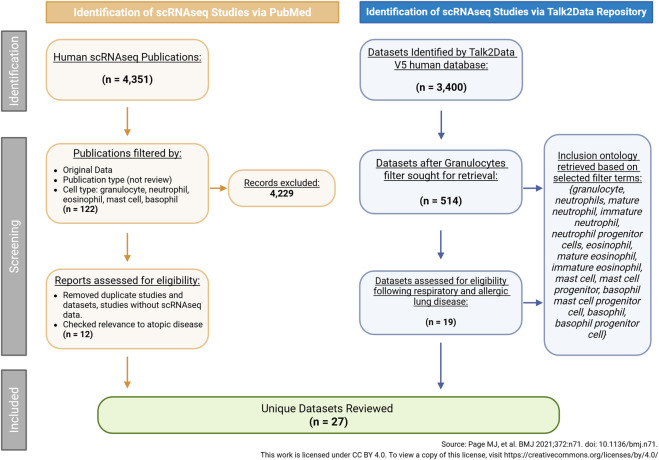
Pubmed was searched for all human scRNAseq publications, and then sequentially for each granulocyte cell type, and finaly manually curated for those studies with original scRNAseq data containing granulocytes and relevant to atopic disease (left). The compendium of human datasets in the Bioturing database was similarly queried sequentially for granulocyte datasets, granulocyte cell-type-specific data sets, and further queried for all atopic disease datasets (right).

We performed a systematic review using two complementary approaches to capture all relevant publications (Figure 2). First, we undertook a search of PubMed using the terms “human” and “single cell RNA sequencing” and then respectively “granulocyte”, “neutrophil”, “eosinophil”, “basophil”, and “mast cell” while filtering for studies including data and excluding reviews. We manually curated the resulting studies (current as of July 2025) to select for those which indeed included scRNA-seq datasets and were relevant to atopic disease. We included a study of chronic obstructive pulmonary disease (COPD) and cystic fibrosis (CF). While these are not atopic diseases, we included these studies for context on the roles of granulocytes in airways disease and because of the emerging role of Type 2 inflammation in a significant proportion of patients with COPD and Cystic Fibrosis (Beech et al., 2024; Manti et al., 2022).

To complement the PubMed search we systematically queried the Talk2Data platform (BioTuring) to identify publicly available human scRNA-seq datasets relevant to granulocytes in atopic and respiratory disease. From the full human compendium (204.9 million cells), we applied hierarchical inclusion criteria: (i) a lineage prefilter to myeloid/granulocyte cells, yielding ∼2.7 million cells from 735 studies; (ii) cell-type filters for canonical granulocyte populations, neutrophils, eosinophils, mast cells, and basophils, within respiratory system disorders and lung tissue, resulting in 545,000 cells from 74 studies; and (iii) disease-specific terms (*asthma, eosinophilic esophagitis, rhinitis, chronic rhinitis, chronic rhinosinusitis, atopic dermatitis*), producing a final set of 19 datasets. Exclusions included non-human data, non-scRNA modalities, off-target cell types, and studies lacking confirmatory disease/tissue metadata or representing duplicates. Automated platform filters were followed by manual verification to ensure consistency and deduplication, and all query terms and counts are reported to support reproducibility. This hierarchical, criteria-driven workflow ensured unbiased and reproducible identification of single-cell granulocyte data across heterogeneous scRNA-seq studies. The complete list of studies meeting either the PubMed or Talk2Data search criteria is provided in Supplementary Table 1.

**FIGURE 3:**
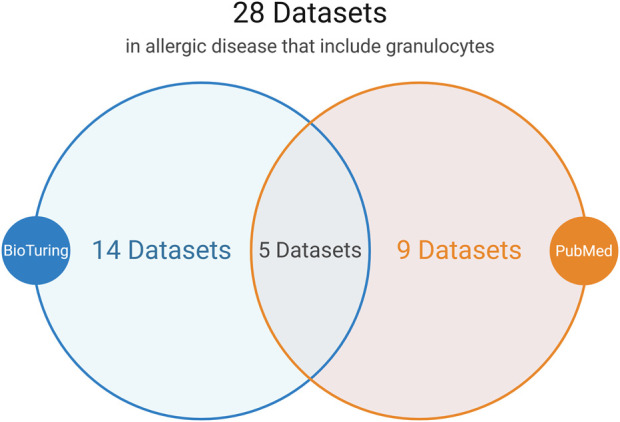
Bioturing and Pubmed Identify Both Overlapping and Distinct Studies. Datasets included in Bioturing but not Pubmed often derived from publications focusing on other cell types but captured some granulocyte data. Studies included in Pubmed but not in Bioturing were either new with data not yet automatically included in the Bioturing database or had data available by direct request to the study authors.

In total, the combined set of data contained in these studies represents 166,759 human granulocyte single-cell transcriptomes in studies of atopic diseases. As shown in Supplementary Table 1, these two differing search methods identified largely unique and complementary sets of studies. We also show the overlap in studies retrieved from these two searches (Figure 3). Common characteristics of PubMed studies not included in Bioturing included recent publication with datasets not yet added to the Bioturing database, and data only available by direct request to authors. Conversely, studies included in Bioturing which did not arise in the Pubmed searches often contained granulocyte data but were focused primarily on other cell types.

Subsequently, we will review key insights gleaned from this work by cell type of interest. In some cases, these studies provided insights into mechanisms of atopic disease, but granulocytes themselves were not the focus of the analysis. We review these studies throughout in the ensuing text according to their relevance to granulocyte biology in allergic disease.

### 2.2 Emerging evidence of high-level phenotypical heterogeneity for neutrophils in atopic diseases

Neutrophils represent the granulocyte population most thoroughly investigated overall in single-cell studies. Returning to the pioneering work by Wigerblad and colleagues on peripheral blood neutrophils, the authors used magnetic selection on the blood of 7 healthy donors and analyzed ∼72,000 neutrophils. They identified four distinct transcriptional subsets in blood, which they interpreted respectively to be immature (Nh0 subset, ∼20% of neutrophils), transitional (Nh1 subset, 57% of neutrophils), low transcriptional activity (Nh2 subset, ∼14% of neutrophils), or enriched for type I interferon-inducible genes (Nh3 subset, ∼7% of neutrophils) (Wigerblad et al., 2022). The association with the interferon-response has been reported in several other publications, supporting the existence of interferon-responsive neutrophils in both peripheral blood and the airways (nasal lavage and bronchoalveolar lavage) (Hatje et al., 2025; Kapellos et al., 2023; Wu et al., 2024; Jayavelu et al., 2025). However, the field has yet to establish the clinical significance of these **interferon-responsive neutrophil** subsets.

In terms of clinical correlation, one notable study, by Yang and colleagues (Yang et al., 2023), describes a patient with acute mixed hypercapnic and hypoxic respiratory failure requiring mechanical ventilation due to asthma exacerbation. Using scRNA-seq at the onset of respiratory failure and during recovery, the authors profiled approximately 9,700 cells, the majority of which were neutrophils. They identified transcriptionally distinct neutrophil subsets that appeared during the resolution phase, characterized by enrichment of metabolic pathways such as the TCA cycle and oxidative phosphorylation. These “**recovery-associated**” neutrophils also expressed transcripts typically linked to macrophage biology, including *CD74* (MHC class II invariant chain), *C1QB* (complement component C1q B chain), and *APOE* (apolipoprotein E). This observation supports the concept of neutrophil heterogeneity and context-dependent plasticity within the inflamed airway.

As highlighted, the context-dependent diversity of neutrophils has also been documented in non-atopic chronic airway diseases, but with emerging links with T2 inflammation. For example, scRNA-seq analysis of sputum from 9 individuals with Cystic Fibrosis (CF) and 5 healthy controls revealed three transcriptionally distinct neutrophil subsets: immature proinflammatory, mature, and heat-shock responsive (Schupp et al., 2020). CF airways were particularly enriched for the immature proinflammatory subset, enriched in transcripts for inflammatory genes such as the chemokine receptor *CXCR4*, *S100A8/A9* (calprotectin), NF-κB-related signaling molecules, while showing reduced expression of maturity (i.e., *FCGR3B* (coding for CD16), and *CXCR2*), phagocytosis-related receptors (e.g., *CD14*, *FCGR2A/3B*), MHC class I molecules, and antigen presentation genes (Schupp et al., 2020). The profile of these neutrophils suggests enhanced inflammatory activation accompanied by diminished antimicrobial cellular functions features associated with the severe bronchiectasis of cystic fibrosis. Notably, CXCR4 and S100A8/A9 upregulation have been observed in murine models of severe asthma, where CXCR4 signaling pathways sustained a pool of **immature-like neutrophils** (Radermecker et al., 2019; Wang et al., 2018). Such parallels between CF and asthma models imply the emergence of a pro-inflammatory, immature neutrophil phenotype as a common feature across chronic airway diseases, potentially contributing to fixed airflow limitation and corticosteroid resistance. Notably, this immature neutrophil phenotype shares transcriptional and functional similarities with the “GRIM” (granule-releasing, immunomodulatory, metabolically active) neutrophils described in 2018 by Forrest and colleagues in their study in which they developed a system designed to recapitulate features of neutrophils in cystic fibrosis (Forrest et al., 2018). Together, several publications now converge toward the existence of a population of **GRIM-neutrophils** characterized by exaggerated proinflammatory gene expression, reduced antimicrobial function, and transcriptional signatures consistent with recent recruitment to inflamed airways.

More recently, Jayavelu and colleagues investigated granulocyte heterogeneity in nasal lavage samples from pediatric patients with asthma (Jayavelu et al., 2025). Using a cell-fixation protocol followed with 3′droplet-based scRNA-seq and a novel computational pipeline to capture low-RNA-content cells, they analyzed 8,281 neutrophils from patients stratified into high- and low-eosinophil groups. Clustering analysis identified seven transcriptionally distinct neutrophil subsets. The most abundant cluster was enriched in the lowEOS group (58.2% vs. 30.7%) and was characterized by high levels of expression of transcripts encoding the cell-surface proteins CXCR1, CXCR2, ITGB2, as well as genes coding for neutrophil extracellular trap (NET) formation and degranulation. The authors suggest that functionally, these cells could potentially contribute to epithelial injury and mucus plugging via the release of elastase, myeloperoxidase, and reactive oxygen species, mechanisms which promote airway obstruction and hyperresponsiveness. In contrast, the second largest cluster, more prevalent in highEOS patients, displayed a strong proinflammatory transcriptional program characterized by elevated expression of IL1B, IL8 (*CXCL8*), and genes involved in TNFα and NF-κB signaling suggesting possible synergistic interactions with eosinophils and thus driving an immunologically distinct type of severe asthma (i.e., T2-high asthma).

Of note, the authors also performed differential gene expression analysis comparing transcriptional signatures between the high and low-EOS groups. They showed higher expression of genes typically associated with eosinophil biology including *IL3RA*, *ALOX5*, and MUC5AC*,* supporting data made several years ago in murine model of asthma, that showed that certain neutrophils exhibit the plasticity to acquire eosinophilic features (Shin et al., 2022). This finding is also supported by a study of adult donors with asthma (Haruna et al., 2024), a study we will return to shortly.

Although, as highlighted by authors, these observations should be validated in larger patient cohorts to determine the reproducibility and clinical relevance of these findings, this study provides interesting evidence that different types of functionally relevant neutrophils contribute to different types of inflammation in the asthmatic airway.

Although COPD is not classified as an atopic disease, recent single-cell analyses have revealed heterogeneous neutrophil states in the COPD airway that correlate with lung function decline, suggesting that neutrophil transcriptional programs may reflect distinct inflammatory endotypes. In a 2023 study by Kapellos and colleagues of early-stage COPD (Kapellos et al., 2023), they identified 3 subsets of neutrophils in bronchoalveolar lavage fluid that they defined as enriched for interleukin signaling (*CXCL8, SOD2, TNFAIP6*), response type I interferon signaling and antiviral defense (*IFITM2/3, ISG15, MX1, OAS1/2*), and neutrophil degranulation and toll-like receptor signaling pathways (*FCGR3B, S100A8/A9,* and *IFITM2*). Given the prevalence of Type 2 inflammation in a subset of patients with COPD, these findings may suggest that neutrophil transcriptional programs, including shared signatures such as interferon-associated responses, could help link airway inflammatory landscape to diseases endotypes.

There are also other studies we identified in our search, which while they do not capture neutrophil single cell transcriptomes, do underscore the relevance of neutrophils to allergic airways disease. For example, the upregulation of neutrophil chemoattractant genes such as *CXCL6* by airway epithelial cells, was shown in a segmental allergen challenge by Alladina and colleagues (Alladina et al., 2023). Conversely, the absence of canonical neutrophil-associated chemokine signatures such as IL8 (*CXCL8*) and the predominance of Type 2 inflammatory signatures stands out in a 2018 publication by Ordovas-Montanes et al. (2018). In their analysis of chronic rhinosinusitis with nasal polyps (CRSwNP), the authors identified mast cells and other myeloid cells expressing Charcot-Leyden crystal (CLC)-associated transcripts and showed that basal progenitor cells retain an “allergic memory” characterized by an aberrant expression of epithelial alarmins TSLP, IL-33, and IL-25 (Ordovas-Montanes et al., 2018). These inflammatory molecules can influence neutrophil recruitment, while antimicrobial secretory functions of inflamed epithelium enables persistent microbial cues that reinforce neutrophil chemotaxis signals. This provides a potential explanation for the chronic, relapsing nature of neutrophil-associated airway diseases.

In summary, while cross-interpretation of published data requires caution due to current methodological limitations, emerging evidence indicates that neutrophils are highly dynamic and context-sensitive cells, exhibiting remarkable transcriptional plasticity. The identification of neutrophils with pro-inflammatory, IFN-responsive, recovery-associated, immature, and “hybrid” molecular features with macrophages or eosinophils reveals a diverse spectrum of transcriptional maturation and activation states, shaped by the local tissue microenvironment. Clinically, this growing understanding reframes neutrophils not as passive bystanders but as active drivers of airway inflammation and remodeling, underscoring the need to further investigate neutrophils at single-cell resolution to resolve their nature in pathogenicity.

### 2.3 Eosinophils in atopic disease

Until recently, single-cell RNA-seq data on eosinophils were limited to murine models, but several recent studies have expanded this field to humans. In 2024, Jorssen, Van Hulst, and colleagues investigated eosinophilopoiesis in human and murine samples using RNA-seq, at single-cell and bulk resolution, as well as high dimensional flow cytometry. They showed that the canonical T_H_2 cytokine IL-5 is not essential for eosinophil differentiation (Jorssen et al., 2024) but for transit progenitor cell amplification. Since around the time of this publication, three additional studies have now been published which examine eosinophils in the context of asthma (Goss et al., 2025; Jayavelu et al., 2025; Haruna et al., 2024).

Haruna and colleagues investigated the potential for granulocyte lineage plasticity, particularly between eosinophils and neutrophils, by performing scRNA-seq on blood from two patients with severe asthma, one atopic individual, and two healthy controls, as well as bone marrow samples from a healthy donor (Haruna et al., 2024). They identified a population of immature metamyelocyte-like neutrophils in severe asthma that co-expressed canonical neutrophil and eosinophil markers (*ELANE*, neutrophil elastase; *MPO*, myeloperoxidase; *PRG2*, major basic protein; *CTSG*, cathepsin G serine protease), suggesting transcriptional overlap between the two granulocytic lineages. They also showed that IL-5 stimulation could drive differentiation of these metamyelocyte-like neutrophils precursors into eosinophils. Further, they identified distinct neutrophil subsets enriched into each donor group: healthy controls (*S100A8/A9*, calprotectin; *FCGR3B*, CD16; *CXCR2*, neutrophil chemokine receptor; *MMP9*, matrix metalloproteinase-9), allergic controls (*CCR1*, chemokine receptor; *CXCR4*, chemokine receptor; *IL1RN*, IL-1 receptor antagonist; *SOCS3*, cytokine signaling regulator), and two asthma-associated clusters. These asthma-associated neutrophils clustered separately from healthy donor neutrophils based on their downregulation of S100A8/A9 and upregulation of inflammatory and innate immune genes (*CXCL8*, IL-8 chemokine; *ICAM1*, intercellular adhesion molecule-1; *TLR2*, Toll-like receptor 2; *NFKBIA*, NF-κB inhibitor α; *PTGS2*, cyclooxygenase-2; *TNFAIP3*, TNF-α–induced protein 3), consistent with increased nitric oxide and bacterial/LPS response pathways. Meanwhile, eosinophils formed a smaller but transcriptionally heterogeneous cluster containing cells from each of the donor populations. Within the bone marrow, eosinophils could be subdivided into two transcriptionally distinct populations resulting from progressive maturation states. The cells were identified based on expression of lineage marker genes such as transcription factors GATA1/2 and XBP1 as well as granule marker genes including *CLC, PRG2*, and *EPX*. One cluster expressed higher levels of granule transcripts and was thus considered to be an immature eosinophil population compared to the other which was considered mature.

Collectively, these findings provide molecular evidence for transcriptional overlap between eosinophil and neutrophil programs, supporting the concept of granulocyte lineage plasticity under inflammatory conditions such as severe asthma.

In their study published in February 2025, Goss et al. sought to leverage scRNA-seq to investigate differential gene expression of blood eosinophils from blood collected amongst 14 subjects, 6 with mild asthma, 4 with severe asthma, and 4 healthy control donors. They isolated and analyzed ∼24,500 eosinophil single-cell transcriptomes and identified three distinct eosinophil sub-clusters. Most eosinophils fell into clusters 1 and 2, while cluster 3, enriched in interferon-responsive genes, contained predominantly severe-asthma–derived cells, suggesting that eosinophils in asthma develop a generally more pro-inflammatory phenotype. Although preliminary, this study showed eosinophil transcriptomic heterogeneity in blood in asthma. We note the presence of an interferon-responsive cluster of eosinophils with interest, given the parallel findings described in the neutrophil literature.

Returning to the publication by Jayavelu et al., eosinophils in the highEOS asthma group expressed genes such as *IL3RA* and *CD69* to a greater degree than in the lowEOS group, as well as *MUC5A*, a gene which has been implicated in mucous plugging, suggesting a more inflammatory or mature state for these eosinophils (Jayavelu et al., 2025). Interestingly, eosinophils (n = 2,450) from the lowEOS group showed expression of genes typically associated with Type 1 and Type 3 inflammation such as *CXCL8*, *IL1B*, and *IL17RA*. Out of 2,450 eosinophils, they identified 4 distinct subsets. The largest cluster was enriched in the highEOS clinical group, while the Eos3 cluster expressed genes often associated with neutrophils including *IL1R2* and *CXCL8* and was enriched in the lowEOS group. Compared with the two eosinophil clusters identified in bone marrow and three in blood in the Haruna and Goss studies, the four clusters resolved here suggest greater transcriptional heterogeneity at the tissue level.

In summary, only a few recent scRNA-seq studies have begun to reveal the transcriptional diversity of human eosinophils, extending beyond murine data. Jorssen et al. (2024) mapped eosinophilopoiesis across species (though their study included bulk RNAseq and single cell proteomic approaches in human cells), while Haruna et al., Goss et al. (2025), and Jayavelu et al. each described distinct eosinophil subclusters in asthma, ranging from IFN-responsive and pro-inflammatory phenotypes to subsets expressing neutrophil-like genes, collectively demonstrating substantial transcriptional heterogeneity and context-dependent plasticity within eosinophils.

### 2.4 Mast cells in atopic disease

**FIGURE 4:**
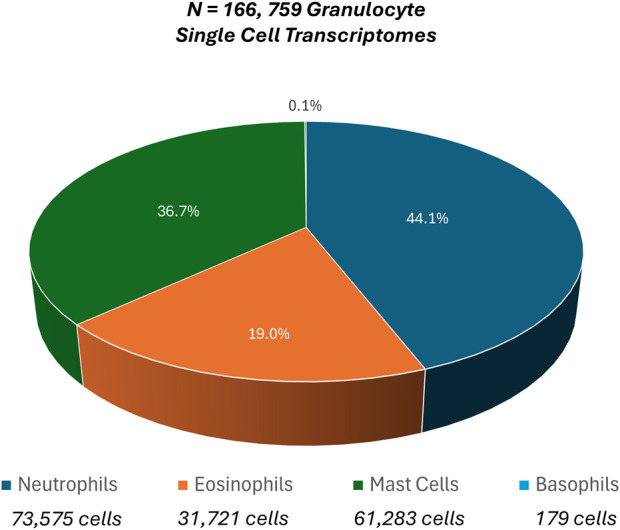
Most Human Granulocyte scRNAseq Data is Comprised of Neutrophils and Mast Cells. In the Bioturing Talk2Data database, the majority of granulocyte single cell transcriptomes come from neutrophils and mast cells. In studies of atopic dermatitis, eosinophilic esophagitis, and any allergic airways disease, this is also true, with neutrophils and mast cells being most numerous, followed by eosinophils and a small number of basophils.

**FIGURE 5:**
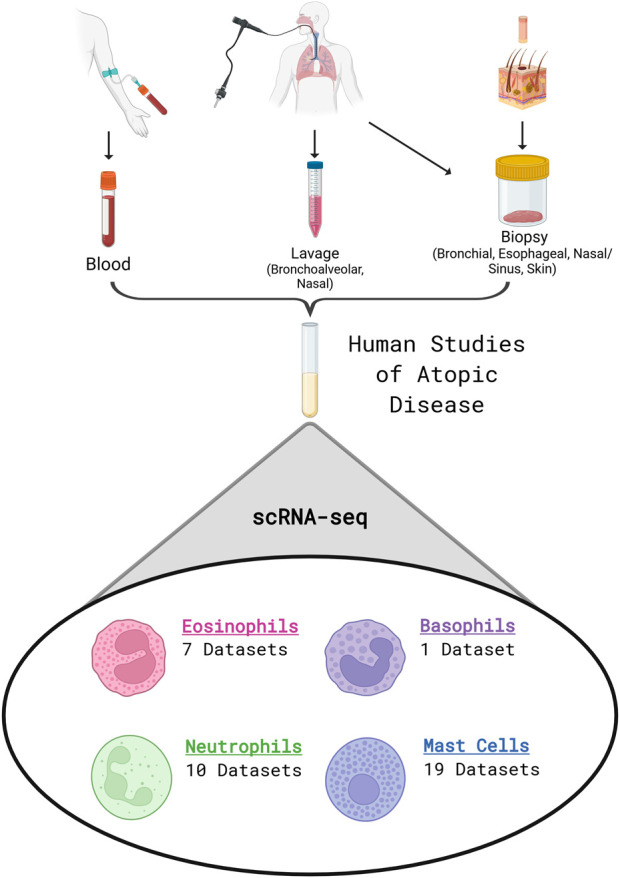
Current Human scRNAseq Data on Atopic Disease Derives from 7 Tissue Sources. Current scRNAseq data in atopic disease spans 7 tissue types including blood, nasal and bronchoalveolar lavage, and endobronchial, esophageal, intranasal/nasal turbinate/sinus, and skin biopsies with studies focused on asthma, eosinophilic esophagitis, atopic dermatitis, and chronic rhinosinusitis with and without nasal polyps. The number of publications containing data for each cell type is displayed.

Mast cells, although not strictly classified as granulocytes, are often grouped with granulocytic cells because of their granule-rich cytoplasm and similar effector functions, and were among the most frequently identified cells in the datasets from studies in the Bioturing database across several disease contexts (Figures 4, 5). Liu and colleagues utilized scRNA-seq to identify transcriptomic differences in inflammatory skin disorders including atopic dermatitis (AD) and psoriasis (Liu et al., 2022). They detected mast cells using *TPSAB1*, gene encoding for the mast cell tryptase stored in their granules, as a marker gene, and the mast cells clustered quite disparately from other detected cell types. They were quantitatively enriched in AD samples. Interestingly, they identified a subset of mast cells enriched in marker genes of mitotic activity. The authors employed droplet-based scRNA-seq in this study. Rochman *et al.* also leveraged droplet-based scRNA-seq in an elegant study assessing transcriptomic changes associated with disease activity in subjects with eosinophilic esophagitis (EoE) (Rochman et al., 2022). They too detected mast cells in their esophageal biopsy samples, again using *TPSAB1* as a marker gene; the mast cell cluster also characterized by expression of genes such as *CPA3* (mast cell granule protease), *LTC4S* (leukotriene synthesis enzyme), *CTSG* (neutrophil serine protease), and *KRT1* (epithelial keratin), and they were enriched in biopsies taken from active disease as compared to disease in remission or healthy control samples.

Meanwhile in the setting of asthma, Siddiqui and colleagues used a segmental airway allergen challenge in a portion of their study on the mechanisms of IL13-induced goblet cell metaplasia, exposing 4 subjects with allergic asthma to house dust mite and 4 to negative control and performing scRNA-seq of cells isolated bronchial brushing samples (Siddiqui et al., 2021). They detected a diverse array of immune cells, and amongst granulocytes detected a small cluster of mast cells as well. In this case, the cluster was contributed to by both the control and house dust mite-treated individuals. These cells were not the focus of the analysis in this case. Alladina et al. also employed a segmental allergen challenge model and characterized mast cells in the asthmatic airway (Alladina et al., 2023). The mast cells were enriched in asthma compared to control and expressed genes related to growth and survival (*IL3RA* [interleukin-3 receptor, CD123], *IL2RA* [interleukin-2 receptor, CD25], *BIRC3* [baculoviral IAP repeat-containing protein 3, an anti-apoptotic regulator], and *BCL2* [B-cell lymphoma 2, an anti-apoptotic protein]), chemotaxis (*CXCR4* [chemokine receptor] and *C5AR1* [complement component 5a receptor 1, CD88]), and they were more transcriptionally active, suggesting proliferation and *in situ* survival. In a recent scRNA-seq analysis of sputum from healthy controls and individuals with asthma, Yan and colleagues identified a discrete mast cell cluster within their dataset (Yan et al., 2025) characterized by expression of similar canonical mast cell genes as in other tissue types including two mast-cell specific tryptase genes, *TPSAB1, TPSB1*, and KIT encoding the mast cell canonical cell surface marker.

Mast cells have also been characterized in nasal polyp tissue in both aspirin-exacerbated respiratory disease and chronic rhinosinusitis with nasal polyps (Ordovas-Montanes et al., 2018; Bangert et al., 2022; Dwyer et al., 2021). In the former, the study by Ordovas-Montanes, mast cells were shown to be key expressers of Type 2 cytokine genes *IL5* and *IL13* as well as the hematopoietic prostaglandin D synthase gene (*HPGDS*). Mast cells have been categorized in the subepithelial space by expression of tryptase only (MCT) or the expression of both tryptase and chymase (MCTC), and Dwyer and colleagues utilized flow cytometry and scRNA-seq to assess the transcriptional correlates of these states. They identified an intermediate expanded population of mast cells which highly expresses CD38 and CD117 and are enriched for markers of proliferation. They identified these cells in pulmonary fibrosis and asthma datasets as well, suggesting a broader role in these other disease states. In the latter study by Bangert and colleagues, three distinct mast cell clusters were identified termed MC1 through MC3. MC1 expressed gene coding for chymase (*CMA1*) and cathepsin G (*CTSG*), while MC2 and MC3 were characterized by lower expression of *KIT* and *FCER1A* (chain of the high-affinity IgE receptor).

Overall, these studies demonstrate that mast cells can be detected in a variety of tissues using droplet-based scRNA-seq, and there is a general trend towards increased transcriptional activity and production of Type 2 mediators by mast cells in samples taken from atopic disease as compared to control samples. However, in each of these studies the relative number of mast cells detected is low, with most of the cells isolated being non-granulocyte myeloid, lymphoid, or non-immune lineages such as epithelial cells. It is therefore not possible to draw conclusions as to disease-specific mast cell phenotypes or to fully assess the effects of donor-to-donor variability on results based on currently available data.

### 2.5 Basophils in atopic disease

There is a paucity of basophil scRNA-seq data in the atopic disease literature. However, the study of human basophils at the single cell level is an area of active research interest, and scRNA-seq data on this rare cell type is beginning to emerge. One important example of such work was reported by Qi and colleagues in 2021 (Qi et al., 2021). The authors used Cellular Indexing of Transcriptomes and Epitopes by Sequencing (CITE-seq) profiling to identify granulocyte lineages from whole blood and bone marrow and showed good correlation with data generated by Cytometry by Time-Of-Flight (Bendall et al., 2011) (CyTOF), a high-dimensional single-cell proteomic profiling method. They performed scRNA-seq using both droplet-based (10X Genomics) and microwell-based (BD Rhapsody) methods, and similarly to two later studies we reviewed (Ben-Baruch Morgenstern et al., 2024; Janssen et al., 2025), found improved detection of granulocyte transcripts using the microwell-based method. The authors showed basophils to be characterized by expression of gene coding for CLC (like eosinophils) as well as the genes *FCER1A* (high-affinity IgE receptor α chain), *GATA2* (GATA-binding transcription factor 2), and *HDC* (histidine decarboxylase). In 2025, Papavasileiou and colleagues also reported on the single cell transcriptome of human basophils (Papavasileiou et al., 2025). The authors also used CITE-seq to identify basophils amongst a population defined by flow cytometry gating as of side scatter_low_, negative for markers of mature hematopoietic lineages (CD3, CD19, CD20, CD14, CD16, CD56, CD235a), expressing CCR3 and FcεRI cells and performed scRNA-seq. Like Qi and colleagues, basophils expressed *CLC, HDC*, and *FCERIA* and also expressed *IL3RA, CCR3*, and *ENPP3*, encoding for the ectonucleotide pyrophosphatase/phosphodiesterase 3 surface molecule (CD203c), known to be associated with basophil activation (Boumiza et al., 2003). Interestingly, they identified two populations, termed Basophils 1 and Basophils 2. Higher expression of *CLC* and *FTL* (encoding ferritin light chain) distinguished the Basophils 2 population. The authors also used a CITE-seq panel of 140 antibody-conjugated oligonucleotides to assess cell surface epitopes between the two transcriptionally defined basophil subsets and found similar expression of these markers in both groups. This study provides novel evidence for basophil transcriptomic heterogeneity, and like the work from Qi and colleagues, underscores the importance of investigating multiple methodological pipelines in the study of granulocytes at the single-cell level.

While basophils were not the focus of any studies of atopic diseases we identified, Jayavelu *et al.* annotate basophils in their dataset, and their results offer some initial insights into airway basophil transcriptional patterns in asthma. The group observed that of the 179 basophils captured in their study, in the high-EOS group the cells upregulated the JAK/STAT signaling pathway, while in the low-EOS group, basophils expressed genes related to type 1 immunity and genes associated with neutrophilic inflammation such as *CXCR1* and *CXCR2*. Together, the results of these few studies are in keeping with results for other granulocyte cell types in that they reveal that basophils are not a uniform effector population. Instead, basophils too appear to adopt context-dependent transcriptional programs that may contribute to the heterogeneity of asthma endotypes, with potential implications for disease severity, corticosteroid responsiveness, and targeted therapy (e.g., JAK inhibitors or cytokine blockade).

## 3 Future directions

Recent years have brought major advances in granulocyte transcriptomics, including in atopic diseases (Figure 5). Once considered elusive and uniform, granulocytes are now recognized as exhibiting distinct transcriptomic states shaped by tissue origin and disease context. Despite this progress, the field remains in its early stages, with several key gaps to address.

First, there is a continued need to address technical hurdles, and to systematize practices for the isolation and processing of these “touchy” cell types. Only then will controlled cross-study comparisons be truly possible. To this end, improved bioinformatic integration of larger datasets will also be crucial for generating more unified insights. Second, existing studies are generally limited to small cohorts of samples, limiting statistical analysis and correlation of “subsets” with clinical features of importance to the population studied, such as disease severity or treatment efficacy. Future studies with larger donor cohorts are essential to disentangle inter-individual variation and link granulocyte subsets to clinical phenotypes. Such efforts will hold clear translational potential, paving the way for new biomarkers and therapeutic targets.

Regarding therapies, the expanding use of monoclonal antibody therapies targeting IgE and cytokine or alarmin pathways (IL-5, IL-4, IL-13, TSLP) has transformed treatment for asthma, chronic rhinosinusitis with nasal polyps, atopic dermatitis, eosinophilic esophagitis, and hypereosinophilic syndromes. However, we are only beginning to understand how these biologics reshape granulocyte transcriptional states. Early evidence suggests pathway modulation, for example, reduced NF-κB activity in myeloid and lymphoid cells from patients treated with mepolizumab or dupilumab (Luo et al., 2024; Diaz-Cabrera et al., 2024). Ongoing and upcoming studies, including Phase 4 trials utilizing benralizumab and dupilumab in moderate-to-severe asthma, will further clarify these effects.

Finally, the transcriptome represents just one dimension of granulocyte biology. Integrating proteomic, spatial transcriptomic, and eQTL mapping approaches with scRNA-seq will provide a more complete understanding of granulocyte function in atopic disease.

### 3.1 Forming a unified understanding of granulocyte transcriptomic signatures in health and disease

As single cell transcriptomics of human granulocytes becomes more feasible and data accumulates from an increasing number of donors, an important goal will be to define the spectrum of granulocyte transcriptomic heterogeneity both in the state of health and to identify patterns of gene expression or even granulocyte subsets which are truly *specific* to individual diseases. This worthy goal remains on the horizon in case of allergic diseases such as asthma and chronic rhinosinusitis, but the field is moving closer. In the studies reviewed here, there are compelling examples of granulocyte heterogeneity and perhaps plasticity. In asthma for instance, Jayavelu and colleagues noted a correlation in gene expression between neutrophils and eosinophils in their lowEOS group. Also in asthma, Haruna et al. noted an expanded population of immature neutrophils which co-expressed neutrophil and eosinophil markers. Meanwhile, Goss et al. noted enrichment for interferon-responsive genes more prevalently in eosinophils derived from severe asthma, a finding which also echoes the discovery of neutrophil subsets which similarly upregulate interferon response genes. These examples give clues that granulocytes may indeed preferentially upregulate specific inflammatory pathways in diseases like asthma. This provides great motivation to rigorously test whether such signatures correlate with atopic disease endotypes or could reveal new endotypes and novel therapeutic targets in the future.

## 4 Conclusions

In summary, granulocytes are key effector cells in a range of atopic conditions, and advances in scRNA-seq are beginning to shed light on their transcriptional heterogeneity in these diseases. Despite advances, the field remains challenged by a lack of a single “best practice” towards employing single cell transcriptomics in these difficult-to-study cells. Moreover, studies employing more individuals and a greater variety of tissue types are needed to help us truly define “atopic” versus “healthy” transcriptional signatures in these cells, but existing data at least support the notion that specific granulocyte subsets may in fact be key players in disease pathophysiology, rather than the entire class of cell types. In the near future, multi-omics studies coupling scRNAseq with other methods such as ATAC-seq, proteomic techniques, and spatial transcriptomics offer the promise of more comprehensive mechanistic insights. There remains much to learn, but the study of the pathophysiology of atopic diseases through the lens of granulocyte biology sits on an exciting frontier.
